# Bicuspid aortic valve phenotype and aortic disease: a magnetic resonance study

**DOI:** 10.1186/1532-429X-13-S1-P208

**Published:** 2011-02-02

**Authors:** David W Fitz, James C Carr, Edwin Wu

**Affiliations:** 1 Northwestern University Feinberg School of Medicine, Chicago, IL, USA

## Objective

The aim of this study was to uncover any association between bicuspid aortic valve (BAV) phenotype and aortic valve disease or aortic dilation.

## Background

BAV is the most common congenital heart defect in the general population, particularly in patients requiring valve surgery. Recent studies have shown cardiovascular magnetic resonance (CMR) to be an accurate and sensitive modality for classifying BAV phenotypes.

## Methods

Patients were selected retrospectively from the database at NMH according to IRB-approved protocols. We identified and selected 217 patients who had a diagnosis of BAV and underwent CMR angiography between 2007 and 2010. For each subject, age, gender, functional state of the BAV, pattern of fusion, aortic root diameter, mid ascending aortic diameter, proximal and distal aortic arch diameter, and primary indication at the time of the MRA were extracted from reports.

## Results

We identified four valve morphologies: type 1, fusion of the right and left cusps (n=152); type 2, fusion of the right and non-coronary cusps (n=48); type 3, fusion of the left and non-coronary cusps (n=9); and unicuspid, two fusions (n=8). We further characterized each type by the number of sinuses, two or three, and the presence or absence of a raphe. Comparing the three types, two sinuses were associated with a significantly higher (P<0.001) proportion among type 2 and type 3 BAV. Type 2 BAV was associated with moderate to severe stenosis (P=0.049) and a larger diameter of the mid ascending aorta (P=0.029). There was no significant difference between aortic regurgitation and aortic root diameter. Tables 1 and 2.

**Table 1:** Pattern of bicuspid aortic valve phenotype

	Type 1 (n= 152)	Type 2 (n=48)	Type 3 (n=9)	Unicuspid (n=8)
3 sinuses w/ raphe	119 (78.3%)	21 (43.8%)	2 (22.2%)	8 (100%)
3 sinuses w/ fusion	8 (5.3%)	6 (12.5%)	0	0
2 sinuses	25 (16.4%)	21 (43.8%)	7 (77.8%)	0

**Table 2:** Clinical characteristics of single-study BAV patients

	Type 1 (n=108)	Type 2 (n=33)	Type 3 (n=8)
Aortic Stenosis			
None	75 (69.4%)	14 (42.4%)	7 (87.5%)
Mild - mod	7 (6.5%)	6 (18.2%)	1 (12.5%)
Moderate	8 (7.4%)	4 (12.1%)	0
Mod - severe	18 (16.7%)	9 (27.2%)	0

Aortic Regurgitation			
None/trace	36 (30.6%)	13 (39.3%)	5 (62.5%)
Mild - mod	48 (44.4%)	12 (36.4%)	2 (25.0%)
Moderate	15 (13.9%)	7 (21.1%)	0
Mod - severe	9 (8.3%)	1 (2.9%)	1 (12.5%)

Aortic Dilation (cm)			
Root	3.99±0.58(2.7-5.7)	4.10±0.67(2.9-5.7)	4.04±0.70(3.0-5.2)
MAA	3.91±0.60(2.0-5.4)	4.08±0.78(2.8-5.8)	3.37±0.58(2.6-4.1)

## Conclusion

CMR is a capable imaging tool for characterizing BAV phenotype. Type 1 BAV is the most frequent phenotype. Two sinus valves are more common among type 2 and type 3 phenotypes. Type 2 is associated with moderate to severe aortic stenosis and a larger mid-ascending aortic diameter.

